# Pro-Angiogenesis Role of LINC00662 From Esophageal Squamous Cell Carcinoma Cells-Derived Extracellular Vehicles

**DOI:** 10.3389/fbioe.2022.772514

**Published:** 2022-04-01

**Authors:** Feng Li, Ren Niu, ShaoLin Gao, FangChao Zhao, Zefang Dong, Hao Zhang, Shujun Li

**Affiliations:** ^1^ The Second Hospital of Hebei Medical University, Shijiazhuang, China; ^2^ Department of Oncology, The Second Hospital of Hebei Medical University, Shijiazhuang, China; ^3^ Department of Thoracic Surgery, The Second Hospital of Hebei Medical University, Shijiazhuang, China; ^4^ Institute of Precision Medicine and Pathology, Jinan University, Guangzhou, China

**Keywords:** esophageal squamous cell carcinoma, extracellular vehicles, LINC00662, microRNA-195-5p, vascular endothelial growth factor a

## Abstract

**Objective:** LINC00662 is oncogenic in some human cancers, but no much was revealed concerning to its specific action in tumor angiogenesis. Given that, our study investigated the role of LINC00662 from esophageal squamous cell carcinoma (ESCC) cells-derived extracellular vehicles (EVs) in angiogenesis through microRNA (miR)-195-5p/vascular endothelial growth factor A (VEGFA) axis.

**Methods:** Clinical tissue samples were collected from patients with ESCC, in which LINC00662, miR-195-5p and VEGFA expression was analyzed. ESCC cells were transfected, from which EVs were isolated. Human umbilical vein endothelial cells (HUVECs) were co-cultured with the pretreated EVs. After that, viability, colony formation ability, invasion, migration and tube formation ability of HUVECs were observed. Tumor xenograft in nude mice was performed to detect the effect of LINC00662, miR-195-5p or EV specific inhibitor GW4869 on tumor development.

**Results:** LINC00662 and VEGFA were upregulated while miR-195-5p was downregulated in the cancer tissue of patients with ESCC. EVs derived from ESCC cells promoted viability, colony formation ability, invasion and tube formation ability of HUVECs. Downregulation of LINC00662 or upregulation of miR-195-5p reversed the promotion of EVs derived from ESCC cells on the viability, colony formation ability, invasion and tube formation ability of HUVECs *in vitro* and *in vivo*. VEGFA overexpression reversed EVs carrying restored miR-195-5p induced effects on HUVECs *in vitro*.

**Conclusion:** In summary, elevated LINC00662 transferred by ESCC cells-derived EVs induces angiogenesis through downregulating miR-195-5p and upregulating VEGFA.

## Introduction

Esophageal squamous cell carcinoma (ESCC) is the main subtype of esophageal cancer (EC) which originates from the stratified squamous epithelium of the esophagus (Reichenbach et al., 2019). Because of repeated inflammation and mucosal damage of the esophagus, it is most often diagnosed by tissue biopsy during upper gastrointestinal endoscopy (Batra et al., 2019). ESCC mostly happens in late adulthood, and patients often experience symptoms such as dysphagia and weight loss (Lam, 2020). For advanced ESCC, cytotoxic agents (docetaxel, cisplatin and 5-FU), molecular-targeting therapy and immunotherapy are systematically applied to palliate symptoms and improve survival (Hirano and Kato, 2019). In tumor progression, tumor cells secrete high levels of pro-angiogenic factors, which contribute to the formation of abnormal vascular networks, leading to poor tumor perfusion (Viallard and Larrivée, 2017). Considering that, fighting against tumor angiogenesis may offer alternative therapeutic targets.

Extracellular vesicles (EVs) is a collective term involving in many subtypes of cell-released, membranous structures, named as exosomes, ectosomes, oncosomes, microvesicles, microparticles, apoptotic bodies, as well as many other names (Théry et al., 2018). EV are cell membrane-derived vesicles, and cancer cells-derived EVs exert multiple roles in malignant progression, including metastasis and angiogenesis (Knox et al., 2020). Hypoxia-conditioned EVs from ESCC cells have been reported to induce tube formation of human umbilical vein endothelial cells (HUVECs) (Mao et al., 2019). As effective carriers, EVs can transfer nucleic acids including miRNA and long noncoding RNA into specific target cells, regulating cell function and tumor microenvironment (Lin et al., 2019). More and more studies have confirmed the pro-angiogenesis effect of cancer-derived EVs carrying tumor-promoting cargos (Lang et al., 2017; Zeng et al., 2018; He et al., 2019). LINC00662 refers to an oncogene in many cancers, which acts notoriously in the fields of tumor metastasis (Cheng B et al., 2020) and radioresistance (Chen et al., 2020). In ESCC, a report has described specifically the pro-metastatic property of LINC00662 (Zhang et al., 2020), but little was discovered in relation to LINC00662-mediated angiogenesis. miR-195-5p is an anti-angiogenic modulator in human cancers (Cai et al., 2018; Dai et al., 2019). In the setting of ESCC, miR-195-5p has been identified as a novel biomarker for cancer diagnosis, classification and prognosis (Li C. Y et al., 2019), but miR-195-5p-dependent angiogenesis remains unknown. Tumor angiogenesis mainly relies on the response driven by vascular endothelial growth factor A (VEGFA) (Claesson-Welsh and Welsh, 2013). VEGFA is commonly amplified in esophageal adenocarcinomas (Cancer Genome Atlas Research, Network et al., 2017) and regulation of VEGFA by miRNAs serves a therapeutic option for EC (Kong et al., 2016; Shen W et al., 2020). For reasons of treatments, our research mainly discussed the effect of LINC00662 delivered by ESCC cells-derived EVs on angiogenesis through modification of miR-195-5p and VEGFA.

## Methods and Materials

### Tissue Collection

A total of 84 ESCC patients (44–79 years old, mean age of 62.02 ± 9.49 years) were recruited from The Second Hospital of Hebei Medical University. Among them, 42 cases were in stage I, 28 cases in stage II, and 14 cases in stage III. All patients underwent tumor resection before chemotherapy. ESCC tissues and adjacent normal tissues were collected and preserved at −80°C. All patients were pathologically diagnosed as ESCC, and no radiotherapy or chemotherapy before surgery was performed. Patients who died from diseases unrelated to ESCC during the follow-up were excluded (He et al., 2020).

### Cell Culture and Transfection

Human normal esophageal epithelial cells (HEEC) and ESCC cell lines EC9706, Eca109, TE-13, TE-1 and TTN were provided by China Center for Type Culture Collection (Wuhan, China). Cells (3 × 10^5^ cells/well) with 80% confluence were subjected to transfection using Lipofectamine 2000 (Invitrogen, CA, United States). Overexpressed (oe)-VEGFA, small interfering RNA (si)-LINC00662, oe-LINC00662, miR-195-5p mimic and their corresponding negative control (NC) were all from GenePharma (Shanghai, China) (Li Z et al., 2019).

### EVs Collection and Identification

ESCC cells were placed in Roswell Park Memorial Institute (RPMI) 1640 medium containing EVs-free 10% fetal bovine serum (FBS) and cultured for 3 d. Then, the cell supernatant was collected and centrifuged to remove cell debris. The EVs were extracted using Hieff™ Quick exosome isolation kit (41201ES50, YEASEN, Shanghai, China). The supernatant and exosome separation reagent were added into the Eppendorf (EP) tube with a proportion of 2:1 overnight, and then centrifuged at 100,00 g for 1–2 h and the precipitate was resuspended in phosphate buffered saline (PBS). The EV suspension (30 μl) together with an equal volume of radioimmunoprecipitation assay (RIPA) lysis buffer was continuously dissolved in microwave (2 times, 10 s/time). Then, the EVs were purified at 4°C at 12,000 × g for 2 min, and the supernatant was retained and stored at −80°C. Protein quantification of EV was performed by bicinchoninic acid (BCA) kit (Beyotime, Nantong, China).

EVs identification: Transmission electron microscope (TEM; JEM-1010, JEOL, Tokyo, Japan) was adopted to observe EV morphology, tunable resistive pulse sensing (TRPS) to examine concentration and diameter of EVs, and Western blot analysis to test EV markers (CD81 and CD9) and non-marker protein (Calnexin and GM130) (Li Z et al., 2019).

### EVs Uptake

EVs uptake was monitored using PKH26 fluorescent labeling kit (Sigma-Aldrich, CA, United States). EV sample was diluted in diluent C, and then PKH26 dye was added and kept at room temperature. After 4 min incubation avoiding light exposure, the reaction was neutralized by 5% bovine serum albumin. Then, EVs, resuspended in PBS were centrifuged at 100,000 g for 70 min, and incubated with HUVECs for 4 h. Subsequently, HUVECs (ATCC, VA, United States) were fixed with 4% paraformaldehyde, labeled with 4′,6-diamidino-2-phenylindole (0.5 μg/ml; Invitrogen) and imaged under a confocal microscope (Carl Zeiss, Oberkochen, Germany) (Zhang et al., 2021).

When HUVECs reached 50–60% confluence, they were incubated with dextran 498 (Dx498, 100 μg/ml) for 2 h to label cell membrane lysosomes, washed with PBS and cultured with complete culture medium for 18 h. Finally, HUVECs were incubated with PKH26-labeled EVs for colocalization analysis.

### Co-Culture of HUVECs and EVs

HUVECs were allocated into groups: PBS group (HUVECs without any treatment), EV group (HUVECs co-cultured with EV from ESCC cells), si-NC-EV group (HUVECs co-cultured with EVs from si-NC-modified ESCC cells), si-LINC00662-EV group (HUVECs co-cultured with EVs from si-LINC00662-modified ESCC cells), mimic-NC-EV group (HUVECs co-cultured with EVs from miR-195-5p mimic NC-modified ESCC cells), miR-195-5p mimic-EV group (HUVECs co-cultured with EVs from miR-195-5p mimic-modified ESCC cells), si-NC group (HUVECs transfected with si-NC), si-LINC00662 group (HUVECs transfected with si-LINC00662), oe-NC group (HUVECs transfected with oe-NC), oe-LINC0066 group (HUVECs transfected with oe-LINC0066), miR-195-5p mimic-EV + oe-NC group (HUVECs co-cultured with EVs from miR-195-5p mimic-modified ESCC cells and further transfected with oe-NC), miR-195-5p mimic-EV + oe-VEGFA (HUVECs co-cultured with EVs from miR-195-5p mimic-modified ESCC cells and further transfected with oe-VEGFA).

### 3-(4, 5-Dimethylthiazol-2-yl)-2, 5-Diphenyltetrazolium Bromide (MTT) Assay

HUVECs were added with 5 mg/ml MTT solution (20 μl, Solarbio, Beijing, China), and cell viability was measured after 12, 24, and 36 h. Using a microplate spectrophotometer (BioTek Instruments, VT, United States), the absorbance at 570 nm was measured (You et al., 2021).

### Colony Formation Test

HUVECs were cultured in RPMI 1640 medium supplemented with 10% FBS, during which the medium was changed every 48 h. After 14 d, visible colonies were fixed with ethanol, stained with 0.1% crystal violet and manually counted.

### Angiogenesis Test

Matrigel (BD Biosciences) was spread over 24-well plates at 100 ml/well and polymerized. HUVECs (resuspended in FBS-free medium) were transferred to the plates at 1 × 10^5^ cells/well and cultured for 6 h. Angiogenesis quantification was judged by the number of branch points of the formed tube in at least five fields of view (Zhou et al., 2019).

### Transwell Assay

HUVECs were resuspended in serum-free medium, and the resuspension (1 × 10^5^ cells/mL, 200 μl) was added to the top side of Matrigel-coated Transwell chamber (Matrigel was not used to examine cell migration). RPMI 1640 medium (600 μl) containing 20% FBS was dispersed in the basolateral chamber. After 48 h of culture, cells were fixed with 4% paraformaldehyde, stained with crystal violet and observed under an optical microscope in five fields of view (Li Z et al., 2019).

### Dual Luciferase Reporter Gene Assay

LINC00662-Wt, LINC00662-Mut, VEGFA-Wt and VEGFA-Mut were cloned and merged with psiCHECK-2 vector (Promega, WI, United States). Employing Lipofectamine 2,000, the vectors were co-transfected with mimic-NC or miR-195-5p mimic into cells and detected by the dual luciferase reporter gene detection system (Promega) (Liu and Meng, 2020).

### RNA-Pull Down Assay

Cells were transfected with 50 nM biotin-labeled Wt-bio-miR-195-5p and Mut-bio-miR-195-5p for 48 h, and subjected to reaction in a specific lysis buffer (Ambion, Texas, United States) for 10 min. The lysate was incubated with M-280 streptavidin magnetic beads (Sigma) pre-coated with RNase-free BSA and yeast tRNA (TRNABAK-RO, Sigma). The beads were incubated at 4°C for 3 h and eluted, and the bound RNA was purified by Trizol, and the LINC00662 enrichment was detected by reverse transcription quantitative polymerase chain reaction (RT-qPCR).

### RIP Assay

The binding of LINC00662/miR-195-5p to AGO2 protein was analyzed by Magna RIP kit (Millipore, United States). Cells were lysed with an equal volume of RIPA Lysis Buffer (P0013B, Beyotime) in an ice bath for 5 min and centrifuged at 14,000 rpm, 4°C for 10 min. The magnetic beads (50 µl) were resuspended in 100 µl RIP Wash Buffer, and added 5 µg antibody AGO2 (1:50, Abcam) or IgG (1:100, Abcam). The magnetic bead-antibody complex was resuspended in 900 µL RIP Wash Buffer and incubated with 100 μl cell lysate overnight at 4°C. The resulting complex was digested with proteinase K and RNA was extracted for PCR detection.

### RT-qPCR

Total RNA in cells and tissues was extracted by Trizol (Takara, Dalian, China) and expression of miR-195-5p was tested by qRT-PCR with the All-in-One miRNA qRT-PCR Detection Kit (GeneCopoeia) and All-in-One miRNA qPCR Primer (GeneCopoeia). Expression of LINC00662 and VEGFA was determined by qRT-PCR RevertAid First Strand cDNA Synthesis Kit (Fermentas) and SYBR Premix Ex Taq (TaKaRa). Primer sequences (Supplementary Table S1) were synthesized by Genepharma. Target expression was analyzed using ABI 7500 (ABI, CA, United States), and standardized by U6 or glyceraldehyde phosphate dehydrogenase. Determination of gene expression was dependent on 2^−ΔΔCt^ method (Li Z et al., 2019).

### Western Blot Assay

Total protein was separated with RIPA lysis buffer (Beyotime) and quantified by BCA kit (Pierce, MA, United States). After separation by 10% sodium dodecyl sulphate polyacrylamide gel electrophoresis (Beyotime), the protein sample was electroblotted onto nitrocellulose membrane (HTS112 M), covered with 5% skim milk and kept overnight with primary antibodies VEGFA (1:1,000), CD81 (1 μg/ml), CD9 (1:1,000), Calnexin (1 μg/ml), GM130 (1 μg/ml) and GAPDH (1:1,000, all from Abcam). Next, horseradish peroxidase-conjugated secondary antibodies goat anti-mouse IgG (1:5,000) and goat anti-rabbit IgG (1:5,000, both from Abcam) were incubated with the membrane, after which signals were detected using SuperSig-nal-West Pico chemiluminescent substrate (Thermo Fisher Scientific, MA, United States). Data analysis was carried out with Image Lab™ 3.0 (Bio-Rad, CA, United States) (Sui et al., 2021).

### Tumor Xenografts in Nude Mice

Eca109 cells (2 × 106) were injected subcutaneously into the hind limb of BALB/c nude mice (6 weeks old, athymic) for the establishment of the ESCC xenograft model. When the nude mice generated the tumors (100 mm^3^), 10 μg EVs from ESCC cells that had been transfected with si-NC, si-LINC00662, mimic-NC, miR-195-5p mimic, PBS, and exosome release inhibitor (GW4869, 1 mg/kg) were subsequently injected into the center of tumor sites every 2 days. Nude mice were euthanized 4 weeks later, and the subcutaneous tumors were obtained to calculate tumor volume (maximum diameter × vertical diameter^2^/2).

### Statistical Analysis

GraphPad Prism 6.0 (GraphPad Software Inc.) and SPSS 20.0 (IBM, NY, United States) were used for data analysis. Values were expressed as mean ± standard deviation and compared by *t*-test (two groups) or analysis of variance (multiple groups). *p* < 0.05 was statistically significant.

## Results

### High LINC00662 and VEGFA and Low miR-195-5p in Cancer Tissues of Patients With ESCC

**FIGURE 1:**
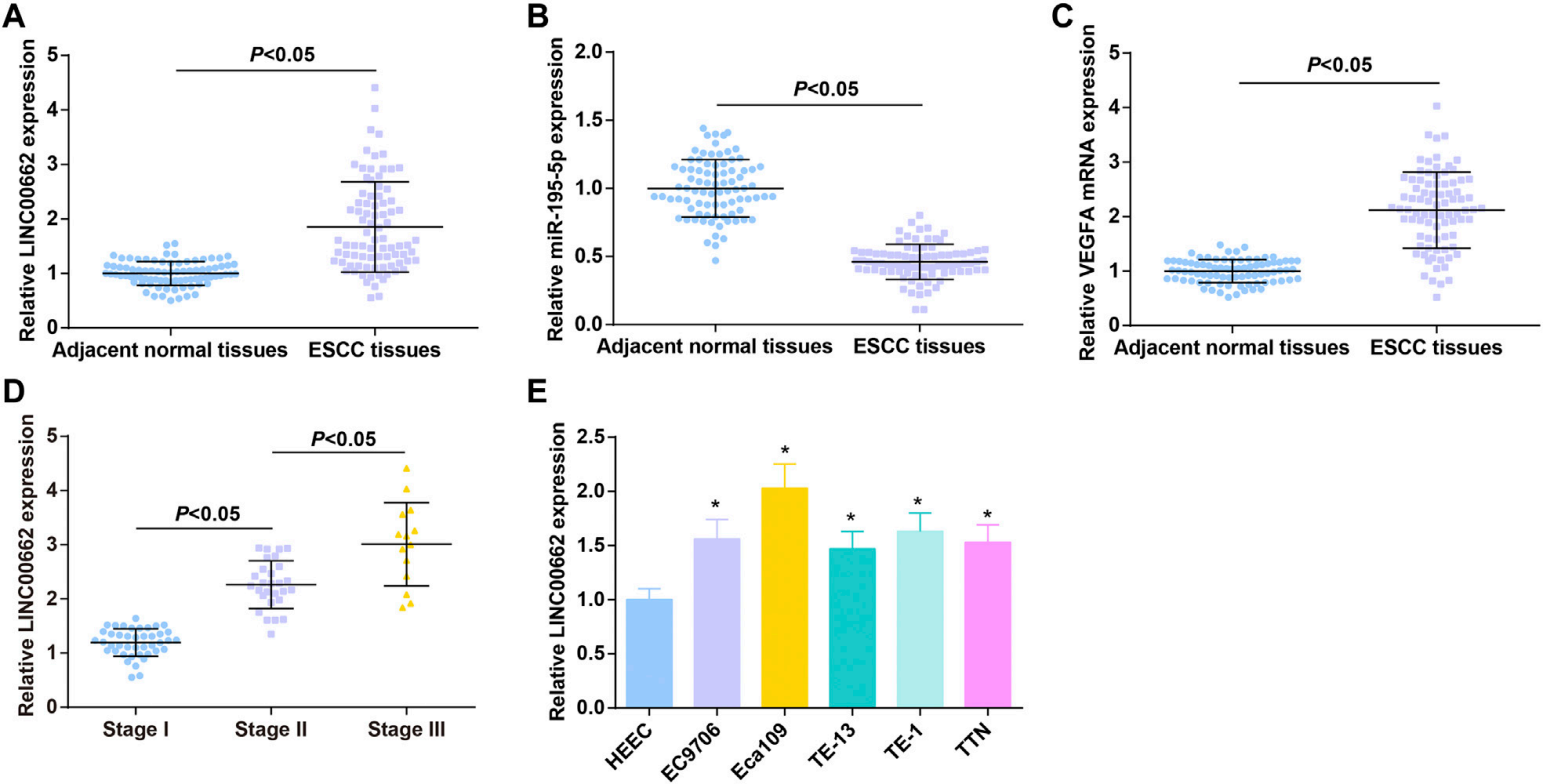
High LINC00662 and VEGFA and low miR-195-5p in cancer tissues of patients with ESCC. **(A–C)**. RT-qPCR detection of LINC00662, miR-195-5p, VEGFA expression in ESCC tissues and adjacent normal tissues of patients; **(D)** RT-qPCR detection of LINC00662 expression in ESCC patients at different TNM stages; **(E)** RT-qPCR detection of LINC00662 expression in HEEC and ESCC cell lines. * *p* < 0.05 vs. HEEC cells.

LINC00662, miR-195-5p, and VEGFA levels in tissue specimens of 84 patients with ESCC were determined by RT-qPCR, as the results reflecting that LINC00662 and VEGFA were upregulated and miR-195-5p was downregulated in ESCC tissues (Figures 1A–C).

ESCC patients were grouped according to TNM staging, and RT-qPCR analysis found higher LINC00662 expression in patients in the late stage than in the early stage (Figure 1D). LINC00662 expression was compared in HEEC and ESCC cell lines (EC9706, Eca109, TE13, TE1 and TTN) by RT-qPCR, and the outcomes indicated that LINC00662 was abundantly expressed in ESCC cell lines (Figure 1E). Because Eca109 had the highest LINC00662 level, it was selected for subsequent experiments.

### Repressive Role of LINC00662 Downregulation in Angiogenesis in ESCC, and Upregulation of LINC00662 has the Opposite Effect

**FIGURE 2:**
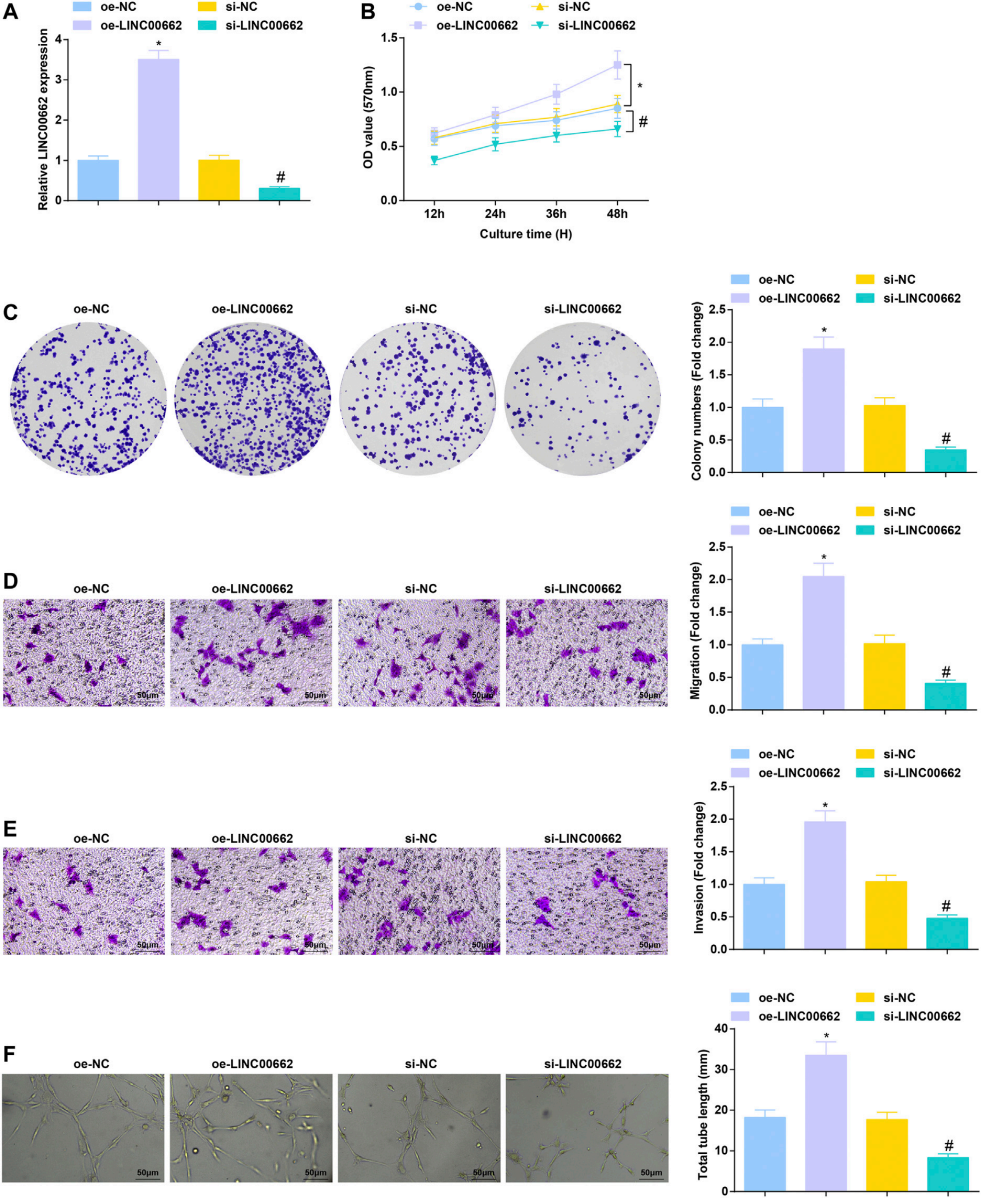
Repressive role of LINC00662 downregulation in angiogenesis. **(A)** RT-qPCR detection of LINC00662 expression in HUVECs after transfection with silenced LINC00662; **(B,C)** Viability and colony formation ability of HUVECs after transfection with silenced LINC00662; **(D,E)** Migration and invasion of HUVECs after transfection with silenced LINC00662; **(F)** Angiogenesis of HUVECs after transfection with silenced LINC00662. * *p* < 0.05 vs. the oe-NC group; # *p* < 0.05 vs. the si-NC group.

To clarify the effect of LINC00662 on Eca109 angiogenesis *in vitro*, LINC00662 was overexpressed or specifically interfered. RT-qPCR test results showed that oe-LINC00662 effectively promoted LINC00662 expression, while si-LINC00662 down-regulated LINC00662 expression in HUVECs (Figure 2A). As reflected by MTT assay, colony formation assay, Transwell assay and angiogenesis test, downregulated LINC00662 depressed proliferation, invasion, migration and angiogenesis but up-regulated LINC00662 promoted these phenotypes of HUVECs (Figures 2B–F). Collectively, LINC00662 downregulation inhibits tumor angiogenesis.

### EVs Collection and Identification

**FIGURE 3:**
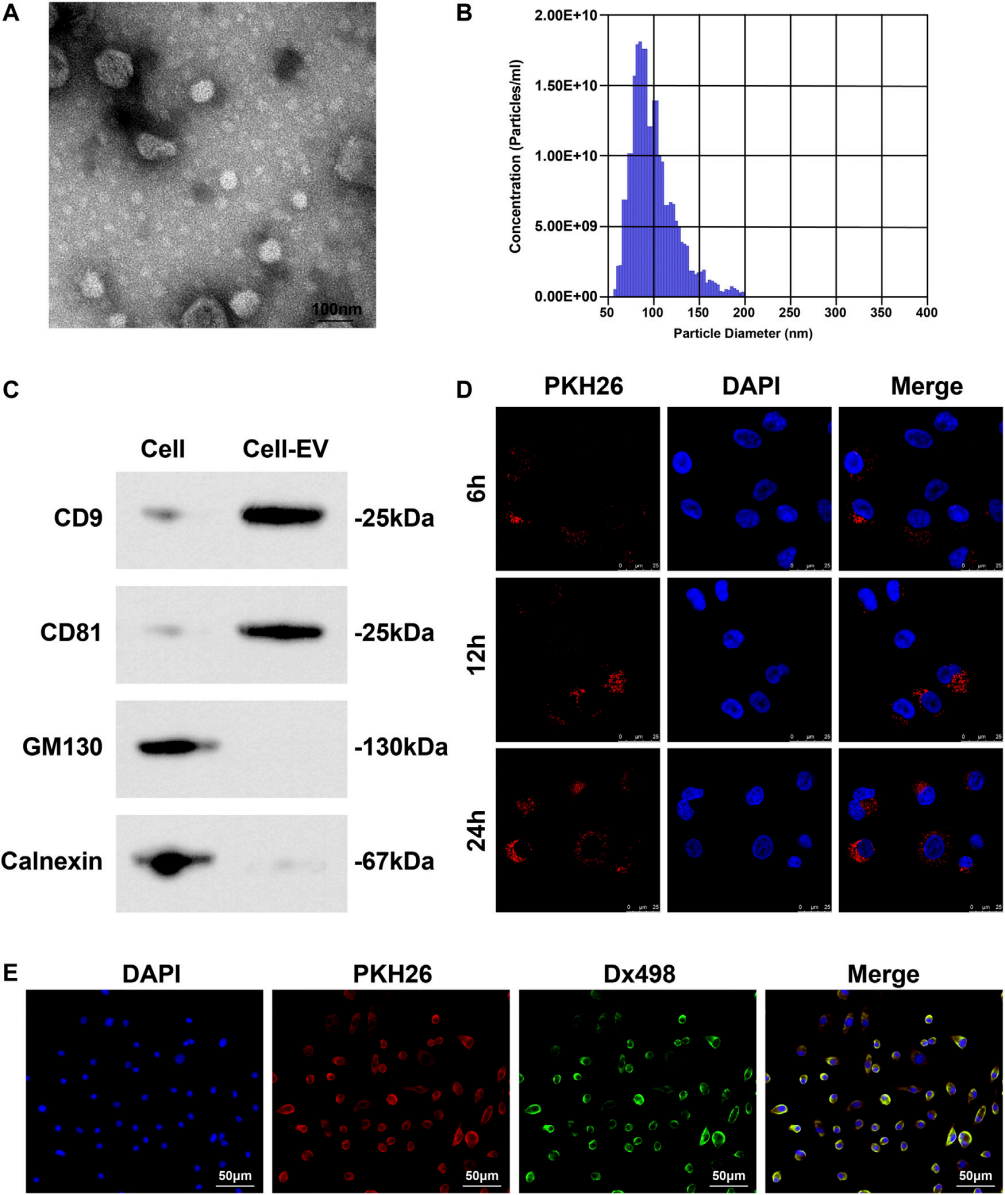
EVs collection and identification. **(A)** Morphology and structure of EVs under TEM; **(B)** Concentration and diameter of EVs examined by TRPS; **(C)** EV markers (CD81, CD9, Calnexin and GM130) analyzed by Western blot; **(D)** Distribution of PKH26-labled EVs in HUVECs; **(E)** Co-localization of lysosome and EVs in HUVECs.

A white precipitate was separated from the culture medium of Eca109 cells by differential centrifugation. Under the TEM, the white precipitate was round or elliptical vesicles with a double-layer membrane structure and a diameter of 40–100 nm (Figure 3A); TRPS showed that the mode of particle diameter was 90 nm and the average diameter was 130 nm (Figure 3B); Western blot detected that CD81 and CD9 were both positively expressed whilst calnexin and GM130 were not expressed in these vesicles (Figure 3C), confirming that EVs were extracted successfully.

PKH26-labeled EVs were co-cultured with HUVECs. Observed under a fluorescence microscope, EVs could enter HUVECs, and over time, the more EVs taken up by HUVECs (Figure 3D). Fluorescence colocalization experiment showed that HUVECs incubated with Dx498 were co-cultured with PKH26-labeled EVs, and EVs and lysosomes were colocalized (Figure 3E).

### Anti-Angiogenesis Effect of EVs-Mediated Delivery of Inhibited LINC00662

**FIGURE 4:**
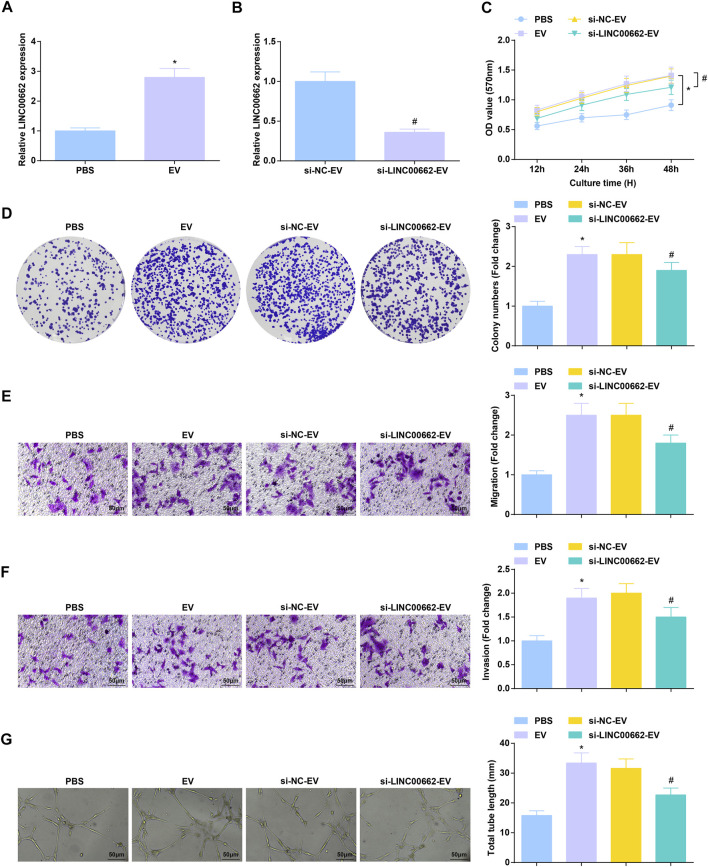
Anti-angiogenesis effect of EVs transfer of inhibited LINC00662. **(A,B)** RT-qPCR detection of LINC00662 expression in HUVECs after co-culture with EVs; **(C,D)** Viability and colony formation ability of HUVECs after co-culture with EVs; **(E,F)** Migration and invasion of HUVECs after co-culture with EVs; **(G)** Angiogenesis of HUVECs after co-culture with EVs. * *p* < 0.05 vs. the PBS group; #*p* < 0.05 vs. the si-NC-EV group.

After co-culture with Eca109 cells-derived EVs, RT-qPCR detection found that LINC00662 expression was increased in HUVECs, while LINC00662 expression was decreased after co-culture with EVs extracted from Eca109 cells transfected with si-LINC00662 (Figures 4A,B). Subsequently, analysis of cell functions revealed that Eca109 cells-derived EVs augmented proliferation, invasion, migration and angiogenesis of HUVECs, while downregulated LINC00662 reversed the effects of Eca109 cells-derived EVs (Figures 4C–G). In conclusion, Eca109 cells-derived EVs transfer of silenced LINC00662 blocks angiogenesis.

### Anti-Angiogenic Role of EVs Carrying Upregulated miR-195-5p

**FIGURE 5:**
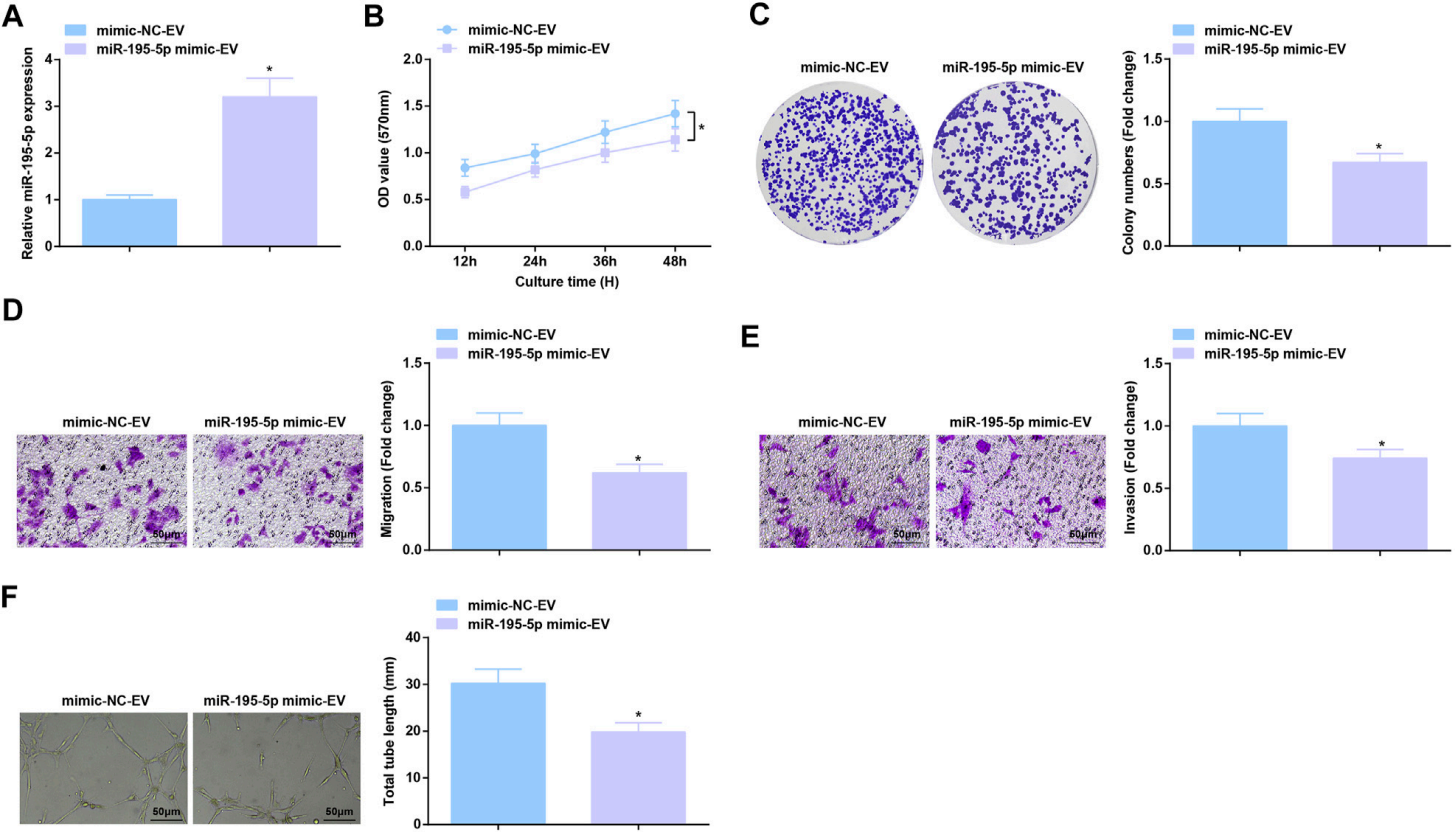
Anti-angiogenic role of EVs expressing upregulated miR-195-5p. **(A)** RT-qPCR detection of miR-195-5p expression in HUVECs after co-culture with EVs; **(B,C)** Viability and colony formation ability of HUVECs after co-culture with EVs; **(D,E)** Migration and invasion of HUVECs after co-culture with EVs; **(F)** Angiogenesis of HUVECs after co-culture with EVs. * *p* < 0.05 vs. the mimic-NC-EV group.

RT-qPCR test measured that co-culture with EVa from miR-195-5p mimic-treated Eca109 cells augmented miR-195-5p expression in HUVECs (Figure 5A). As a result of miR-195-5p upregulation, Eca109 cells-derived EVs-induced aggressive behaviors and angiogenesis of HUVECs were all constrained (Figures 5B–F). In short, EVa transfer of upregulated miR-195-5p exerts anti-angiogenic effect.

### Binding Relation Between LINC00662 and miR-195-5p; Targeting Relation Between miR-195-5p and VEGFA

**FIGURE 6:**
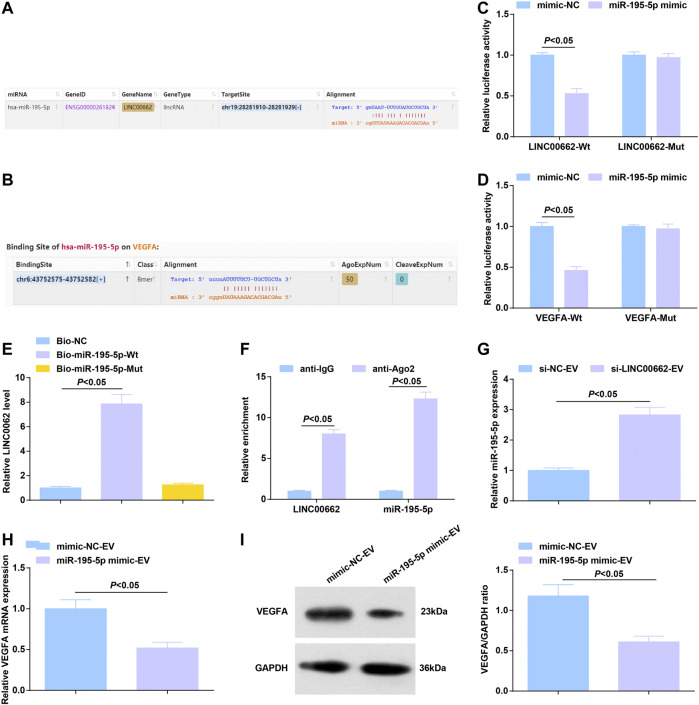
Binding relation between LINC00662 and miR-195-5p; targeting relation between miR-195-5p and VEGFA. **(A,B)** Binding sites of LINC00662 and miR-195-5p, miR-195-5p and VEGFA; **(C,D)** Dual luciferase report results; **(E)** RNA pull-down test detected the enrichment of LINC00662; **(F)** RIP detected the binding of LINC00662 and miR-195-5p to AGO2; **(G)** RT-qPCR detection of miR-195–5p expression in HUVECs after co-culture with EVs; **(H,I)** RT-qPCR and Western blot analysis of VEGFA mRNA and protein expression in HUVECs after co-culture with EVs.

On the bioinformatics website, a binding relationship was predicted between LINC00662 and miR-195-5p (Figure 6A), as well as miR-195-5p and VEGFA mRNA (Figure 6B). Using dual luciferase reporter assay, it was estimated that miR-195-5p mimic reduced the luciferase activity of LINC00662-Wt and that of VEGFA-Wt (Figures 6C,D). RNA-pull down assay showed that Mut-bio-miR-195-5p had no significant effect on LINC00662 enrichment and Wt-bio-miR-195-5p could increase the amount of LINC00662 enrichment (Figure 6E). RIP experiment suggested an elevation in Ago2-bound LINC00662, indicating that LINC00662 could directly bind with miR-195-5p (Figure 6F).

Alternations of gene expression were observed: EVs delivery of silenced LINC00662 elevated miR-195-5p expression in HUVECs (Figure 6G) while that of upregulated miR-195-5p lowered VEGFA levels (Figures 6H,I).

### Reversal of miR-195-5p-Mediated Effects on HUVECs by Overexpressed VEGFA

**FIGURE 7:**
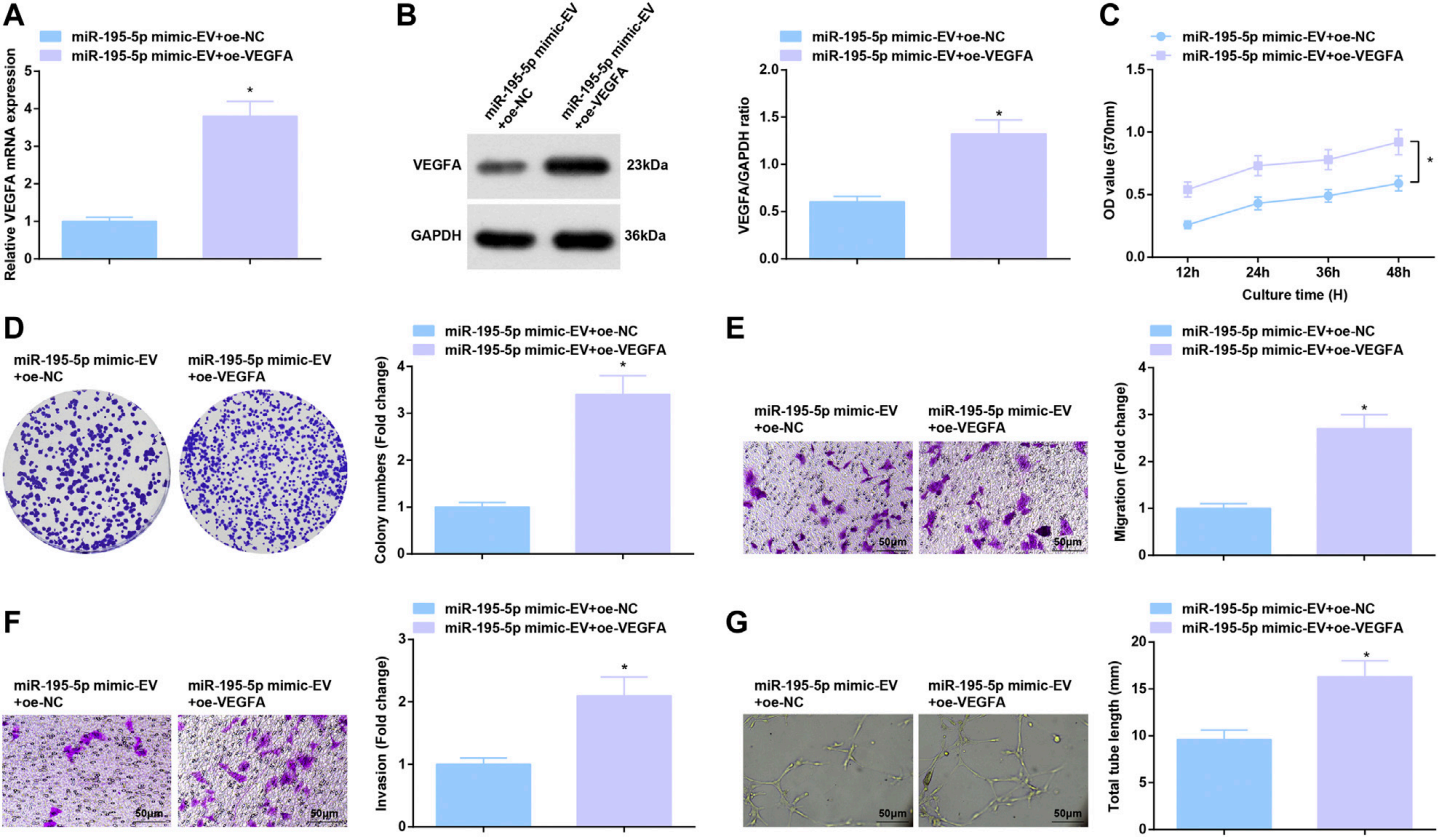
Reversal of miR-195-5p-mediated effects on HUVECs by overexpressed VEGFA. **(A,B)** RT-qPCR and Western blot analysis of VEGFA expression in HUVECs after co-culture with EVs and transfection with overexpressed VEGFA; **(C,D)** Viability and colony formation ability of HUVECs after co-culture with EVs and transfection with overexpressed VEGFA; **(E,F)** Migration and invasion of HUVECs after co-culture with EVs and transfection with overexpressed VEGFA; **(G)** Angiogenesis of HUVECs after co-culture with EVs and transfection with overexpressed VEGFA. * *p* < 0.05 vs. the miR-195-5p mimic-EV + oe-NC group.

miR-195-5p/VEGFA axis-induced function for HUVECs was further evaluated. After co-culture with EVs carrying upregulated miR-195-5p, HUVECs were transfected with oe-VEGFA, which successfully elevated VEGFA expression (Figures 7A,B). Overexpression of VEGFA reversed the effects of EVs carrying upregulated miR-195-5p on proliferation, invasion, migration and angiogenesis of HUVECs (Figures 7C–G).

### Tumor Suppression Effect of EVs Carrying Downregulated LINC00662 or Upregulated miR-195-5p in Mice

**FIGURE 8:**
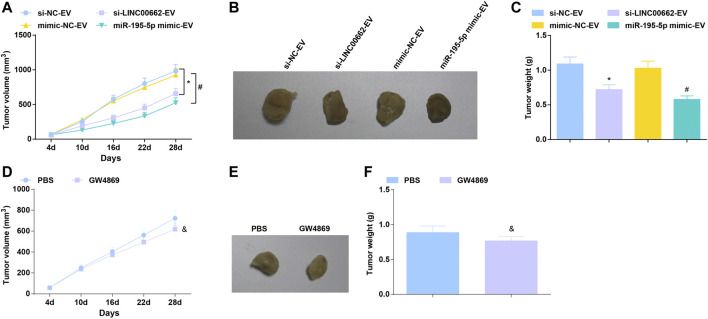
Tumor suppression effect of EVs expressing downregulated LINC00662 or upregulated miR-195-5p. **(A,D)** Tumor volume changes in nude mice; **(B,E)**. Representative tumors in nude mice; **(C,F)** Tumor weight in nude mice. * *p* < 0.05 vs. the si-NC-EV; #*p* < 0.05 vs. the mimic-NC-EV group; & *p* < 0.05 vs. the PBS group.

To further determine the angiogenic role of EVs in vivo, an ESCC tumor-bearing nude mice model was established and treated with EVs. In mice xenograft model, after treatment with EVs delivering depleted LINC00662 or restored miR-195-5p, tumors grew slowly, accompanied by smaller tumor volume and lighter tumor weight (Figures 8A–C). Furthermore, the effect of EV-specific inhibitor GW4869 on the growth of nude mice was observed, reflecting that GW4869 treatment had a better efficacy to repress tumor growth (Figures 8D–F). Simply, LINC00662 suppression or miR-195-5p overexpression inhibits the growth of tumor.

## Discussion

**FIGURE 9:**
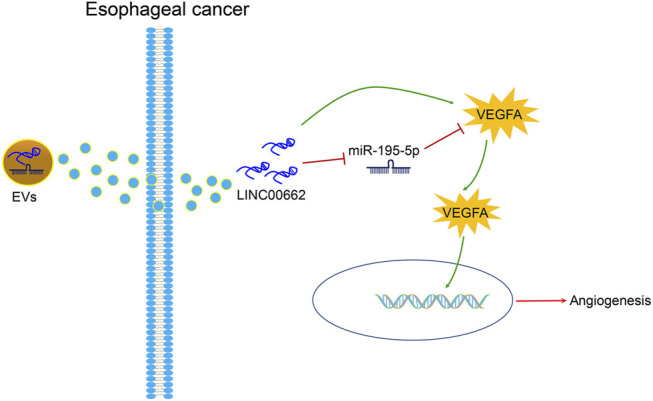
ESCC cells-derived EVs transferal of up-regulation of LINC00662 to promote angiogenesis in esophageal cancer by downregulating miR-195-5p and upregulating VEGFA.

In the tumor microenvironment in EC, induction of angiogenesis is pro-tumorigenic, and its management is critical to the development of anti-angiogenic therapy (Barzi and Lenz, 2012; Lin et al., 2016). In the present article, it was clearly described that silenced LINC00662 transfer by ESCC cells-derived EVs attenuates angiogenesis by miR-195-5p-dependent VEGFA downregulation (Figure 9).

To begin with, LINC00662 expression was found to be heightened in cancer tissues of patients with ESCC and was associated with advanced TNM stage. Consistently, LINC00662 high expression has been examined in cancer tissues of patients with colorectal cancer, indicating a positive correlation with tumor T stage (Wang et al., 2019). Elevation of LINC00662 has also been measured in patients with ESCC, which is negatively correlated with patients’ clinical outcome (Zhang et al., 2020). For HUVECs, knocking down LINC00662 made relives of proliferation, invasion, migration and tube formation abilities, confirming that LINC00662 silencing possesses anti-angiogenic effect. Bo Cheng *et al.* have displayed that LINC00662 overexpression increases proliferation, invasion and migration of colon cancer cells *in vitro* and tumor growth *in vivo* (Cheng B et al., 2020). In addition, a report by Long Cheng *et al.* has implicated the inhibitory role of LINC00662 knockdown as to proliferation, migration and invasion of breast cancer cells (Cheng L et al., 2020). At present, very few analysis has evaluated the complete mechanism of LINC00662-mediated angiogenesis.

Afterwards, ESCC cells-derived EVs were found to augment angiogenesis, but inhibited LINC00662, effectively transported by EVs could attenuate angiogenesis *in vitro* and tumor formation *in vivo*. In the light of an article composed by Yu Mao *et al.*, ESCC cells-secreted EVs under hypoxia stimulate angiogenesis by inducing HUVECs to proliferate, invade, migrate and form tubes *in vitro* (Mao et al., 2019). In addition to EVs from ESCC cells, EVs from other cancer cells present pivotal actions in angiogenesis. As exampled by a late paper, EVs secreted by hypoxic papillary thyroid cancer cells enhance endothelial tube formation (Wu et al., 2019). On the other hand, EVs extracted from gastric cancer cells could be internalized by vascular endothelial cells, so as to activate angiogenesis and tumor growth (Yang et al., 2018). Nils Ludwig *et al.* have experimentally observed that after internalization of EVs from head and neck squamous cell carcinoma cells by HUVECs, proliferation, migration and tube formation of HUVECs are all promoted (Ludwig et al., 2018). Remarkably, hypoxia-induced EVs from lung cancer cells cause formidable force in accelerating proliferation and tube-forming abilities of HUVECs (Mo et al., 2020).

Subsequently, miR-195-5p, as a downstream actor of LINC00662, attracted our attention. Gene expression measurement found lowly expressed miR-195-5p in ESCC tissues, showing consistency with a former article (Shen S et al., 2020). Next, experimental assays proved that EVs delivery of restored miR-195-5p reduced angiogenesis *in vitro* and tumor growth *in vivo*. Indeed, much efforts have been paid to disclose the anti-angiogenic function of miR-195-5p in cancers, including squamous cell lung cancer cells (Liu et al., 2019), ovarian cancer (Dai et al., 2019) and prostate cancer (Cai et al., 2018).

It is of interest that miR-195-5p could directly inhibit VEGFA (Maroof et al., 2019), and the finding was echoed with our research. Importantly, data analysis further proved that VEGFA overexpression reversed miR-195-5p-dependent inhibition of angiogenesis. Based on the observation of Ruoqin W *et al.*, the conditioned medium of CRC cells enhances migration, invasion, and tube formation of HUVECs, which could be reversed by VEGFA silencing (Wang et al., 2020). Meanwhile, Lijun L *et al.* have elaborated that in the treatment of non-small cell lung cancer with anlotinib, suppressed VEGFA signal facilitates to restrain the activities of HUVECs (Liang et al., 2019). Accordingly, it has been established that VEGFA overexpression can eliminate the anti-angiogenic effect induced by miR-130 b in prostate cancer (Mu et al., 2020).

Overall, our study analysis obtained that ESCC cells-derived EVs transferal of downregulated LINC00662 decreases miR-195-5p-targeted VEGFA and relives angiogenesis induction. This research helps the development of potential molecule-targeted therapy for angiogenesis in tumor progression.

## Data Availability

The original contributions presented in the study are included in the article/Supplementary Material, further inquiries can be directed to the corresponding author.
