# Supplementary material for: Pro-Angiogenesis Role of LINC00662 From Esophageal Squamous Cell Carcinoma Cells-Derived Extracellular Vehicles
Source: Front Bioeng Biotechnol. 2022 Apr 1;10:772514. doi: 10.3389/fbioe.2022.772514 (PMC9011136; doi:10.3389/fbioe.2022.772514)
Supplement: Supplementary file 1 [file Table1.docx]

**Supplementary Table 1** Primer sequences used in our experiment

| Genes | Forward (5′‐3′) | Reverse (5′‐3′) |
| --- | --- | --- |
| GAPDH | ACCACAGTCCATGCCATCAC | TCCACCACCCT GTTGCTGTA |
| U6 | GCTTCGGCAGCACATATACTAAAAT | CGCTTCACGAATTTGCGTGTCAT |
| miR-195-5p | TAGCAGCACAGAAATATTGGC | Universal primer |
| LINC00662 | TTTGCCTTGTTCCTGAGCTT | CACCTCATGGATGCAGAGAA |
| VEGFA | CCTGTGACTAACGGCTGGAA | ACGCCTGCAGGACTACAATC |

Note: GAPDH, glyceraldehyde-3-phosphate dehydrogenase; miR-195-5p, microRNA-195-5p; LINC00662, long noncoding RNA LINC00662; VEGFA, vascular endothelial growth factor A
